# Effect of the COVID-19 pandemic on the mental health, daily and occupational activities among health professionals in Colombia: a national study

**DOI:** 10.1186/s12888-022-04337-9

**Published:** 2022-11-04

**Authors:** Augusto Peñaranda, Elizabeth García, Lucia C. Pérez-Herrera, Annabelle Trojan, Daniel Peñaranda, Juan Molina, Sergio Moreno-López

**Affiliations:** 1 Otolaryngology Department, Fundación Santa Fe de Bogotá, Avenida 9 No. 116 -20, office 207, Bogotá, 110111 Colombia; 2 School of Medicine, Universidad de Los Andes, Bogotá, Colombia; 3 Otolaryngology and Allergy Research Groups, Unidad Médico Quirúrgica de Otorrinolaringología (UNIMEQ-ORL), Bogotá, Colombia; 4 Allergy Section, Department of Pediatrics, Fundación Santa Fe de Bogotá, Bogotá, Colombia; 5 Instituto de Investigaciones Inmunológicas, Universidad de Cartagena, Cartagena, Colombia; 6 Otolaryngology Section, Fundación Universitaria de Ciencias de la Salud – Hospital de San José, Bogotá, Colombia; 7 Fellow Consultation-Liaison Psychiatry, Universidad El Bosque, Bogotá, Colombia

**Keywords:** Prevalence, Health professionals, COVID-19, Mental health, Pandemic, Associated factors

## Abstract

**Background:**

The COVID-19 pandemic has placed an unprecedented physical and mental burden on healthcare workers who are frequently at high risk of infection, particularly in low-income countries. This study aimed to assess the prevalence and associated factors of anxiety, depression, and stress, as well as changes in daily and occupational activities among healthcare professionals due to the COVID-19 pandemic in Colombia.

**Methods:**

An observational, cross-sectional study was conducted between February and June 2021. The survey incorporated validated mental health tools such as the Generalized Anxiety Disorder–7, the Patient Health Questionnaire-9, and the Perceived Stress Scale-10. Multivariable ordinal logistic regression analysis was performed to determine the factors associated with severe mental health outcomes.

**Results:**

Among 1345 healthcare workers the prevalence of anxiety, depression, and stress were 75.61, 59.18, and 53.09%, respectively. Anxiety (OR:1.44; 95%CI:1.16–1.8), depression (OR:1.74; 95%CI:1.27–2.37), and stress (OR:1.51; 95%CI:1.18–1.94) were more frequent in women, and individuals who expressed fear of a negative outcome (death, sequelae) (OR:2.25; 95%CI:1.60–3.25), (OR:1.49; 95%CI:1.03–2.16) and (OR:2.36; 95%CI:1.69–3.29) respectively. Age was negatively associated with anxiety (OR:0.98; 95%CI:0.98–0.99), stress (OR:0.98; 95%CI:0.97–0.99), and depression (OR:0.97; 95% CI:0.96–0.98). Reduction in consultations and surgeries (OR:1.01; 95%CI:1.0–1.01) was positively associated with anxiety. Due to the pandemic, most specialists expected to incorporate drastic long-term (> 1 year) changes in their clinical setting and daily activities.

**Conclusions:**

The prevalence of anxiety, depression, and stress is higher among Colombian healthcare workers compared to previous reports. Further research regarding these psychological outcomes is needed to achieve early mental health intervention strategies.

**Trial registration:**

Hospital Universitario Fundación Santa Fe, Ethical Committee Registration ID: CCEI-12992-2021.

## Background

Severe acute respiratory syndrome coronavirus 2 (SARS-CoV-2) as the causative agent of COVID-19 has rapidly spread worldwide, drastically disrupting healthcare systems and placing an unprecedented burden on healthcare professionals. By December 2021, the World Health Organization (WHO) has reported over 240 million confirmed cases and nearly 5 million deaths throughout the different waves of the disease, multiple mutations of the virus, and significant differences in clinical patterns and outcomes [1, 2]. In Colombia, up to 6 million cases of COVID-19 and over 133 thousand deaths have been registered, ranking the country as the 11th hardest hit in terms of mortality rate. Although control measures to contain the spread of the disease have been applied and over 6 billion vaccines have now been administered globally, new strains are emerging. During previous severe acute respiratory syndrome outbreaks, disproportionate infection rates among healthcare workers resulting in long-term adverse psychological and occupational outcomes had been reported [3, 4].

Since the spread of COVID-19, Vizheh et al. stated that healthcare specialists reported higher occupational stress levels and higher rates of psychological symptoms [5]. These outcomes could be related to excessive workload, inadequate support, a critical shortage of personal protective equipment, hospital beds, and ventilators [3, 6]. On a more personal level, the restrictions implemented to contain and reduce the risk of infection impacted the regular daily and leisure activities which may also lead to an increased risk of anxiety, depression, burnout syndrome and stigma in healthcare workers [7]. Several studies conducted during the COVID-19 pandemic have reported a decline in mental wellbeing among health care professionals [3, 5, 6]. From June to September 2020, Mental Health America (MHA) applied a survey to describe their experience and reported that 93% of health care workers were experiencing stress, 86% reported anxiety, and 76% reported exhaustion and burnout [8]. Being worried about exposing their child was reported by 76% of participants, and nearly half of them expressed fear of infecting a partner or older adult family member, while 39% of reported inadequate emotional support [8]. In 2021 a systematic review reported the results of 24 studies predominantly from urban China indicating that COVID-19 had a considerable impact on the psychological wellbeing of front-line hospital staff and suggested that nurses may be at higher risk of adverse mental health outcomes [9]. Likewise, in a meta-analysis performed in 2021 with 38 studies, Saragih et al. reported that anxiety, depression, and distress presented a significant rise in healthcare workers during the 2020 COVID-19 pandemic with a pooled prevalence of 40% (95% CI: 29–52%), 37% (95% CI: 29–45%), and 37% (95% CI: 25–50%), respectively [10].

These studies highlight the importance of mental wellness in physicians during times of crisis, yet further studies are needed to capture a broader picture of the situation to efficiently manage future sanitary emergencies and thus prevent long-term impact on front-line staff. To date, very few studies have been published in English regarding these psychological outcomes in Latin America. Regarding the Colombian population, one study reported the effects of the pandemic on the mental health, daily and occupational activities of otolaryngologists, and allergists [11]. A cross-sectional study reported figures that range of 50% for any type of altered mental state [12] and approximately 72% of anxiety symptoms in Colombian physicians [13]. Despite some international studies have performed these analyses using internationally validated questionnaires [14], few studies have applied these tools in Latin American countries. This study aimed to describe the prevalence and associated factors of depression, anxiety, stress, and the changes in daily and occupational activities among Colombian healthcare professionals during the second wave of the COVID-19 pandemic.

## Methods

### Study design

An observational, cross-sectional study was conducted to determine the prevalence and associated factors of anxiety, depression, and stress levels in a group of healthcare professionals during the second wave of the COVID-19 pandemic in Colombia. The study was based on a non-probabilistic, consecutive sampling, using self-administered, anonymous online surveys to collect sociodemographic and mental health data from February 05, 2021, to June 30, 2021. Internationally validated questionnaires such as the Generalized Anxiety Disorder-7 (GAD7), the Patient Health Questionnaire-9 (PHQ9), and the Perceived Stress Scale-10 (PSS10) were used to determine the frequency of anxiety, depression, and stress, respectively. A sociodemographic questionnaire was also applied and assessed information about age, marital status, family income, working status, workload, work income before and after the pandemic, personal protective equipment, and geographic location. Data collection was performed online, and participants were invited to fill out the survey at any time. Ethics Committee of the Hospital Universitario Fundación Santa Fe de Bogotá approved this study (CCEI-12992-2021) according to the Helsinki Declaration, and all methods were performed in accordance with the relevant guidelines and ethic regulations. Moreover, this study was approved and involved the participation of the National Academy of Medicine from Colombia in the dissemination plans of this research. Informed Consent was obtained from all participants. No incentives were offered for study participation.

### Study population

In terms of the eligibility criteria, healthcare workers registered in the National Unified Registry of Human Resources in Health from Colombia and conducted in-person consultations and/or telemedicine were included. Exclusion criteria were specialists who reported a prior diagnosis of mental health disorders confirmed by a psychiatrist or mental health professional, and those who reported any acute/chronic condition that could limit their ability to answer the questionnaires. The sample size was estimated based on a meta-analysis by Pappa et al. that assessed the prevalence of mental health disorders among 33,062 healthcare workers due to the COVID-19 pandemic [15]. A minimum sample size of 250 participants was calculated considering a pooled prevalence of depression of 22.8%, bearing in mind the following formula [16]:

$$n\ge \frac{Z_{1-\frac{\alpha }{2}}^2\ast p\ast \left(1-p\right)}{d^2}$$

A 5% significance and precision level were applied, as well as a 5% adjustment for probable losses. Regarding the sample selection method, a non-probabilistic, consecutive sampling was conducted. Despite the participants registered to do the survey, only the population who completed all the questionnaires was included in the analysis.

### Mental health questionnaires

Symptoms of anxiety, depression, and stress were assessed using validated Spanish versions of the following measurement tools: GAD-7 [17], PHQ-9 [18], and PSS-10 [19]. The GAD-7 scale was used to assess symptoms of anxiety over the past 2 weeks, ranging from 0 to 21 points as follows: normal (0–4), mild (5–9), moderate (10–14), and severe (15–21) anxiety (23). The cutoff point for identifying General Anxiety Disorder was a score of 10 in the GAD-7 questionnaire [17]. The PHQ-9 assesses depression symptoms and includes 9 criteria of the Diagnostic and Statistical Manual of Mental Disorders [18]. Each item is scored from 0 (not at all) to 3 (nearly every day) according to the level of discomfort of the patient [18]. The PHQ-9 ranges between 0 to 27 and can be classified as follows: none (0–4), mild (5–9), moderate (10–14), moderately severe (15–19), severe (20–27). A total score of ≥10 in the PHQ-9 has a sensitivity and a specificity of 88% for major depression [18]. The PSS-10 questionnaire contains 10 items that measure the perception of stressful experiences over the past month. Responses range from 0 (never) to 4 (very often), and the total score can be classified as follows: low stress (0–13), moderate stress (14–26), and high perceived stress (27–40) [20]. For this study, the severity of the symptoms of anxiety, depression, and stress was classified considering the cutoff values of the GAD-7, PHQ-9, and PSS-10 scales.

### Variables related to COVID-19 and daily activities questionnaires

A “Fear score of COVID-19” developed by the researchers of this study was applied using a scale of 1 to 5 to assess fear of contagion, fear of the possibility of a negative outcome (death, negative sequelae), and fear of infecting a family member and/or friends. Moreover, a questionnaire regarding the opinion on when (less than 3 months, in 3 to 12 months, more than 1 year, never again) healthcare workers would expect to engage again in regular daily and leisure activities was applied. This questionnaire was developed by “The New York Times” and previously applied to 511 epidemiologists [21]. We highlight that their answers only reflect their opinion and individual life circumstances and should not be used as guidelines for the public.

### Statistical analysis

Frequencies and percentages were calculated for the quantitative variables. Central tendency, and dispersion measures for the quantitative variables were estimated. The prevalence of symptoms of anxiety, depression, and stress was calculated along with its 95% confidence interval. Bivariate and multivariate analyses were carried out to explore the associations between the levels of anxiety, depression and stress and the sociodemographic and occupational covariates due to the COVID-19 pandemic. These analyses were based on an ordinal logistic regression analysis. The predictors of the model were selected considering the biological plausibility reported by prior studies as primary criteria, and the possible statistical association within the variables. Variables with clinical relevance first, or those with a Fisher or Kruskall Wallis test with a *p*-value ≤0.2 were included in the multivariate analysis. The full, crude, and adjusted models are reported to compare the strength of the associations with depression and to assess the presence of confounding variables in the analysis. Percentage decrease in monthly income was calculated comparing income before the pandemic and during the fieldwork (October–November 2020). Finally, the goodness of fit of the model was assessed and assumptions were verified through a linearity test, proportional odds, and through the estimation of deviance residuals and leverage values. Statistical significance for the multivariate models was established at *p* < 0.05. Statistical analysis was performed using Stata 16MP software. A 5% significance level for the comparisons was established before data collection.

## Results

A total of 1345 individuals were included in this study, of which 39.26% were over the age of 50, 66.17% (*n* = 840) were women, and 46.10% (*n* = 620) were specialist doctors. The baseline demographic characteristics of the study population are described in Table 1. Most of the sample was based in urban Colombia, i.e., Bogotá (*n* = 663), Antioquia (*n* = 118), 66% of the participants were women, and nearly 40% were over the age of 50. Near half of the study population (49,44%) performed face-to-face consultation, while 25.58% worked as telemedicine practitioners. Overall, 19.03% (*n* = 256) of the study population considered that the personal protection elements provided by their employer were not enough to prevent COVID-19 infection. The most frequently used biosafety elements in their practice were surgical masks with 32.94% (*n* = 443), and N95 respirators with 27.43% (*n* = 369).

**Table 1:** Baseline demographic and occupational characteristics of the study population

Variables	Total ***n*** = 1345	
	n	%
**Sex. Female/Male**	890/455	66.17/33.83
**Age in years** ^**(a)**^	47.12 (12.82)	43.01 (36.97–56.07)
**Age group**		261
**30 years-old or less**	143	10.63
**> 30 to 40 years-old**	324	24.09
**> 40 to 50 years-old**	350	26.02
**> 50 to 60 years-old**	337	25.06
**> 60 to 70 years-old**	161	11.97
**70 years-old or more**	30	2.23
**Number of people in the household**		
**1**	109	8.10
**2**	309	22.97
**3**	373	27.73
**4**	385	28.62
**5 or more**	169	12.57
**Marital status**		
**Married**	646	48.03
**Divorced/widowed**	127	9.44
**Single**	372	27.66
**Free union**	200	14.87
**Occupation**		
**Nurse**	102	7.58
**Specialist**	620	46.10
**General physician**	199	14.80
**Dentist/Specialty resident/Therapist**	196	14.57
**Other type of occupation**	228	16.95
**Years of work experience** ^**(a)**^	19.1 (11.3)	20 (10–28)
**Regarding the workload compared to PRE-PANDEMIC months this**		
**has increased**	576	42.83
**has decreased**	390	29.00
**still the same**	379	28.18
**Work mode**		
**Face-to-face consultation**	665	49.44
**Telemedicine**	344	25.58
**Emergency care for no covid-19 patients**	146	10.86
**Emergency care for covid-19 patients**	145	10.78
**ICU**	122	9.07
**Surgical assistant**	62	4.61
**Considers the security elements sufficient**		
**No**	256	19.03
**Provider of biosafety elements**		
**Occupational Risk Manager**	240	17.84
**Company/Institution/Hospital where you work**	612	45.50
**Yourself**	321	23.87
**Biosecurity item delivered**		
**Mask**	443	32.94
**Face mask N95**	369	27.43
**Coat**	452	33.61
**Surgical cap**	286	21.26
**Antifluid elements**	72	5.35
**Surgical gloves**	284	21.12
**Glasses**	322	23.94
**Alcohol or antibacterial gel**	117	8.70
**Percentage reduction in consultation during the pandemic** ^**(a)**^	26.23 (25.9)	20 (0–50)
**Reduction percentage of your income** ^**(a)**^	23.5 (27.78)	20 (0–40)

^(a)^ Values are reported as mean (standard deviation) and median (p25-p75)

### Prevalence of psychological disorders

Table 2 shows the prevalence of depression, anxiety, stress, and burnout in the study population. The frequency of symptoms was established as follows: anxiety (75.61%), depression (59.18%), and stress (53.09%). Overall, the prevalence of anxiety was higher than the frequency of depression or stress. A total of 587 (43.64%) healthcare professionals presented these 3 psychological disorders simultaneously. When considering burnout, the frequency of high-level emotional exhaustion, depersonalization, and low-level personal accomplishment were 34.28, 16.13, and 23.49% respectively.

**Table 2:** Prevalence and severity of depression, anxiety, stress, and conditions relating to Covid-19 pandemic in the study population

Variable	Total		
	(***n*** = 1345)		
	n	%	95% CI
**Anxiety**	1017	75,61	(73,24 - 77,83)
**Depression**	796	59,18	(56,53- 61,77)
**Stress**	714	53,09	(50,41 - 55,73)
**High level Emotional Exhaustion**	461	34,28	(31,79 - 36,85)
**High level Depersonalization**	217	16,13	(-, 14, 18, 19, 26)
**Low level Personal Accomplishment**	316	23,49	(21,30 - 25,83)
**Presence of Depression in combination with**			
**Anxiety**	756	56,21	(53,54 - 58,83)
**Stress**	598	44,46	(41,82 - 47,12)
**Presence of Anxiety in combination with**			
**Stress**	674	50,11	(47,44 - 52,77)
**Presence of Depression in combination with**			
**Anxiety and stress**	587	43,64	(41,01 - 46,30)
**Emotional Exhaustion**		261	
**Low**	616	45,80	–
**Moderate**	268	19,93	–
**High**	461	34,28	–
**Depersonalization**		261	
**Low**	911	67,73	–
**Moderate**	217	16,13	–
**High**	217	16,13	–
**Personal Accomplishment**		261	
**Low**	316	23,49	–
**Moderate**	543	40,37	–
**High**	486	36,13	–
**Factors associated with contagion**		261	
**Travel to areas of virus circulation**	309	22,97	–
**Close contact with a case**	381	28,33	–
**None**	628	46,69	–
**Have you been diagnosed with Covid-19?**			
**Yes**	281	20,89	–
**Have you been isolated on suspicion of contagion with Covid-19?**			
**Yes**	564	41,93	–
**Have you been vaccinated to Covid-19?**			
**Yes**	1140	84,76	–
**Any of the following family members has been diagnosed with Covid-19**			
**Father**	102	7,58	–
**Mother**	115	8,55	–
**Brother/sister**	296	22,01	–
**Son**	178	13,23	–
**Spouse**	191	14,20	–
**Comorbidities**			
**hypothyroidism**	842	62,60	–
**arterial hypertension**	199	14,80	–
**autoimmune disease**	61	4,54	–
**diabetes**	46	3,42	–
**heart disease**	42	3,12	–
**COPD**	18	1,34	–
**Have you been afraid of contagion by Covid-19**			
**Yes**	1183	87,96	–
**Have you been afraid of the possibility of a negative outcome (death, sequelae) due to Covid-19**			
**Yes**	1150	85,50	–
**Have you been afraid of the possibility of infecting your family and / or friends with Covid-19?**			
**Yes**	1247	92,71	–
**Fear score against (On a scale of 1 to 5):**			
**Fear of contagion** ^**(a)**^	4 (3–5)		
**Negative outcome (death, sequelae)** ^**(a)**^	4 (3–5)		–
**Infect a family member** ^**(a)**^	5 (4–5)		–

^(a)^ Values are reported as median (p25-p75)

### Factors associated with severity of anxiety, depression, and stress

Bivariate and multivariate analysis via ordinal logistic regression of the demographic and clinical variables associated to anxiety, depression, and stress levels are shown in Table 3. Anxiety (OR: 1.44; 95% CI: 1.16–1.8.), depression (1.74; 1.27–2.37), and stress (1.51; 1.18–1.94) were more severe in women. Age was negatively associated with anxiety (0.98; 0.98–0.99), stress (0.98; 0.97–0.99), and depression severity (0.97; 0.96–0.98). Higher depression levels were found in single/divorced/ widowed participants (2.3; 1.48–3.59). Higher levels of anxiety and depression were found in participants who reported that their workload increased during the pandemic due to COVID-19 (2.18; 1.67–2.84); (2.07; 1.58–2.71), while higher levels of stress were found in the participants who reported that their workload “remained the same”. Likewise, higher levels of anxiety, depression, and stress were found in participants that expressed fear of the possibility of a negative outcome (death, negative sequelae) due to COVID-19 (2.25; 1.60–3.25), (1.49; 1.03–2.16), and (2.36; 1.69–3.29) respectively. The reduction in the number of consultations and surgery (1.01; 1.001–1.01) was also associated positively with anxiety severity, while the reduction in income during the pandemic was positively associated with higher levels of stress (1.01; 1.001–1.02) and depression (1.01; 1.001–1.02). Moreover, active healthcare workers presented higher levels of anxiety, depression, and stress (1.51; 1.21–1.89), (1.43; 1.14–1.79), and (1.31; 1.02–1.69) respectively. An interaction between diabetes and Covid-19 vaccination had statistically significant effects (0.084; 0.017–0.40) on anxiety levels, while an interaction between female gender and marital status (single/divorced/widowed) had statistically significant effects on depression levels (0.54; 0.32–0.89).

**Table 3:** Factors associated with severity of Anxiety, Depression, and Stress via ordinal logistic model

Variable	Anxiety (GAD 7)						Depression (PHQ 9)						Stress (PSS 10)					
	Multivariate model ^a^			Reduced model ^b.g^			Multivariate model ^c^			Reduced model ^d.g^			Multivariate model ^e^			Reduced model ^f.g^		
	OR	95% CI		OR	95% CI		OR	95% CI		OR	95% CI		OR	95% CI		OR	95% CI	
Sex ^h^																		
Female	1.45	1.16	1.82	**1.44** ^i^	**1.16**	**1.80**	1.47	1.17	1.86	**1.74**	**1.27**	**2.37**	1.40	1.08	1.80	**1.51**	**1.18**	**1.94**
Age in years	0.98	0.97	0.99	**0.98**	**0.98**	**0.99**	0.98	0.97	0.99	**0.98**	**0.97**	**0.99**	0.97	0.96	0.98	**0.97**	**0.96**	**0.98**
Marital status (reference category: married)																		
Single/divorced/widowed	0.89	0.70	1.12	–	–	–	1.43	1.13	1.82	**2.30**	**1.48**	**3.59**	1.48	1.14	1.93	–	–	–
Free union	1.00	0.74	1.35	–	–	–	1.38	1.01	1.88	1.20	0.68	2.14	1.13	0.80	1.59	–	–	–
Are you currently working?																		
Yes	0.84	0.51	1.40	–	–	–	0.64	0.39	1.06	–	–	–	0.70	0.40	1.22	0.64	0.37	1.12
Type of contract																		
Provision of services	0.83	0.67	1.03	–	–	–	0.88	0.71	1.10	0.90	0.72	1.12	0.85	0.67	1.09	0.88	0.69	1.11
Do you work in a face-to-face external consultation?																		
Yes	0.92	0.75	1.14	–	–	–	1.00	0.80	1.24	–	–	–	1.12	0.88	1.43	–	–	–
Do you work at ICU?																		
Yes	1.45	1.02	2.06	–	–	–	1.27	0.89	1.80	1.28	0.90	1.81	0.87	0.58	1.29	0.81	0.55	1.20
Do you work in telehealth?																		
Yes	0.93	0.73	1.17	–	–	–	1.01	0.79	1.28	1.28	0.90	1.81	0.88	0.68	1.15	–	–	–
The workload during the pandemic (reference category “has decreased”)																		
remains the same	1.14	0.85	1.52	1.08	0.82	1.43	1.09	0.81	1.47	1.12	0.84	1.50	1.36	0.98	1.89	**1.99**	**1.48**	**2.69**
has increased	2.26	1.70	2.99	**2.18**	**1.67**	**2.84**	1.94	1.46	2.58	**2.07**	**1.58**	**2.71**	2.06	1.50	2.83	1.32	0.96	1.82
Are you an active healthcare worker?																		
Yes	1.44	1.15	1.81	**1.51**	**1.21**	**1.89**	1.42	1.13	1.78	**1.43**	**1.14**	**1.79**	1.38	1.07	1.78	**1.31**	**1.02**	**1.69**
Have you been diagnosed with SARS-CoV-2?																		
Yes	1.21	0.95	1.56	–	–	–	1.34	1.05	1.72	–	–	–	1.29	0.98	1.71	–	–	–
Do you have a diagnosis of diabetes?																		
Yes	0.56	0.31	1.01	**4.47**	**1.08**	**18.54**	1.08	0.60	1.95	**2.21**	**1.45**	**3.35**	0.71	0.37	1.38	0.70	0.36	1.34
Do you have a diagnosis of arterial hypertension?																		
Yes	1.21	0.89	1.63	–	–	–	1.47	1.09	1.99	–	–	–	0.88	0.62	1.24	–	–	–
Have you been afraid of contagion by Covid-19?																		
Yes	2.24	1.50	3.34	**2.13**	**1.43**	**3.16**	2.25	1.48	3.43	–	–	–	2.17	1.38	3.40	–	–	–
Have you been afraid of the possibility of a negative outcome (death/sequelae) due to Covid-19																		
Yes	2.15	1.48	3.11	**2.25**	**1.6**	**3.25**	1.44	0.99	2.09	**1.49**	**1.03**	**2.16**	1.62	1.08	2.43	**2.36**	**1.69**	**3.29**
Have you been vaccinated against SARS-CoV-2																		
Yes	0.93	0.70	1.24	1.01	0.76	1.35	0.92	0.69	1.24	–	–	–	0.73	0.52	1.01	1.26	0.96	1.66
Percentage reduction in consultation/surgeries during the pandemic	1.00	1.00	1.01	**1.01**	**1.00**	**1.01**	1.00	0.99	1.00	0.99	0.98	1.01	1.00	0.99	1.01	–	–	–
Percentage reduction in income during the pandemic	1.01	1.01	1.02	–	–	–	1.01	1.01	1.02	**1.01**	**1.01**	**1.02**	1.01	1.01	1.02	**1.01**	**1.01**	**1.02**

^a^ Log-likelihood Intercept only: − 1811.062; Log-likelihood Model: -1690.539; AIC: 3427.079; BIC: 3546.774; ^b^ Log-likelihood Model: -1702.342; AIC: 3432.684; BIC: 3505.542; ^c^ Log-likelihood Intercept only: − 1800.648; Log-likelihood Model: -1697.505; AIC: 3443.010; BIC: 3567.909; ^d^ Log-likelihood Model: -1702.266; AIC: 3440.533; BIC: 3534.207; ^e^ Log-likelihood Intercept only: − 1113.446; Log-likelihood Model: -1009.307; AIC: 2062.614; BIC: 2177.105; ^f^ Log-likelihood Model: -1021.314; AIC: 2070.629; BIC: 2143.487; ^g^ The reduced model was based on the Furnival-Wilson leaps-and-bounds algorithm, Brant test *p* > 0.05, link test p < 0.05; ^h^ For dichotomic variables reference category is” no”; ^i^ Bolded numbers highlight the significant associations between the variables

No collinearity problems were found through the linearity and the goodness-of-fit tests, both tests showed good models ‘specification. In addition, the proportional odds assumption was not rejected for all models (*p* values> 0.10). Likewise, no extreme or influential values were found for the residuals and leverage values.

### Variables related to COVID-19

At the time of the study, 20.89% (*n* = 281) of the participants tested positive for SARS-Cov-2 and 41.93% (*n* = 564) had been isolated on suspicion of infection, in contrast, 84.76% (*n* = 1140) had been vaccinated for Covid-19. Around 87.96% (*n* = 1183) reported fear of contagion by SARS-CoV-2, and 85.50% (*n* = 1150) were afraid of the possibility of a negative outcome as death or negative sequelae due to Covid-19 infection. Finally, up to 92.71% (*n* = 1247) reported being afraid of the possibility of infecting their family and friends with Covid-19. These results are shown in Table 2.

### Changes in daily and leisure activities due to COVID-19

Table 4 reports the daily and leisure activities that participants considered they would soon engage in. The activities that this population expected to engage in within the next 3 months included: eating at a restaurant (56.80%), getting a haircut at a salon or barbershop (49.96%), seeing a doctor for a non-urgent appointment (46.32%), and hiking, or picnicking outdoors with friends (40.52%). Conversely, many healthcare professionals expressed that they would never go out again with someone they do not know well (28.62%), workout at a gym or fitness studio (20.52%), ride a subway/bus (15.17%), attend a church or other religious service (11.52%), work in a shared office (8.62%), or attend a wedding or a funeral (8.25%).

**Table 4:** Daily and leisure activities the health workers might start doing soon

Question	***n*** = 1345		Question	***n*** = 1345	
	n	%		n	%
**Attend a sporting event. concert or play**		261	**Ride a subway or a bus**		
**< 3 months**	73	5.43	**< 3 months**	301	22.38
**3 to 6 months**	110	8.18	**3 to 6 months**	78	5.80
**6 to 12 months**	265	19.70	**6 to 12 months**	151	11.23
**> 1 year**	703	52.27	**> 1 year**	372	27.66
**Never again**	121	9.00	**Never again**	204	15.17
**Does not apply**	73	5.43	**Does not apply**	239	17.77
**Attend a wedding or a funeral**			**Travel by airplane**		
**< 3 months**	136	10.11	**< 3 months**	422	31.38
**3 to 6 months**	128	9.52	**3 to 6 months**	205	15.24
**6 to 12 months**	316	23.49	**6 to 12 months**	264	19.63
**> 1 year**	565	42.01	**> 1 year**	341	25.35
**Never again**	111	8.25	**Never again**	64	4.76
**Does not apply**	89	6.62	**Does not apply**	49	3.64
**Attend a small social event or dinner with a small group of people**			**Vacation overnight within driving distance**		
**< 3 months**	483	35.91	**< 3 months**	385	28.62
**3 to 6 months**	254	18.88	**3 to 6 months**	223	16.58
**6 to 12 months**	301	22.38	**6 to 12 months**	258	19.18
**> 1 year**	237	17.62	**> 1 year**	354	26.32
**Never again**	47	3.49	**Never again**	79	5.87
**Does not apply**	23	1.71	**Does not apply**	46	3.42
**See a doctor for a nonurgent appointment**			**Hike or picnic outdoors with friends**		
**< 3 months**	623	46.32	**< 3 months**	545	40.52
**3 to 6 months**	238	17.70	**3 to 6 months**	210	15.61
**6 to 12 months**	183	13.61	**6 to 12 months**	210	15.61
**> 1 year**	189	14.05	**> 1 year**	242	17.99
**Never again**	70	5.20	**Never again**	92	6.84
**Does not apply**	42	3.12	**Does not apply**	46	3.42
**Exercise at a gym or fitness studio**			**Visit elderly relatives or friends in their home**		
**< 3 months**	245	18.22	**< 3 months**	446	33.16
**3 to 6 months**	103	7.66	**3 to 6 months**	215	15.99
**6 to 12 months**	186	13.83	**6 to 12 months**	265	19.70
**> 1 year**	388	28.85	**> 1 year**	296	22.01
**Never again**	276	20.52	**Never again**	67	4.98
**Does not apply**	147	10.93	**Does not apply**	56	4.16
**Get a haircut at a salon or barbershop**			**Send children on play dates**		
**< 3 months**	672	49.96	**< 3 months**	341	25.35
**3 to 6 months**	187	13.90	**3 to 6 months**	123	9.14
**6 to 12 months**	152	11.30	**6 to 12 months**	135	10.04
**> 1 year**	194	14.42	**> 1 year**	131	9.74
**Never again**	76	5.65	**Never again**	46	3.42
**Does not apply**	64	4.76	**Does not apply**	569	42.30
**Eat at a dine-in restaurant**			**Hug or shake hands when greeting a friend**		
**< 3 months**	764	56.80	**< 3 months**	339	25.20
**3 to 6 months**	200	14.87	**3 to 6 months**	148	11.00
**6 to 12 months**	172	12.79	**6 to 12 months**	229	17.03
**> 1 year**	149	11.08	**> 1 year**	485	36.06
**Never again**	28	2.08	**Never again**	113	8.40
**Does not apply**	32	2.38	**Does not apply**	31	2.30
**Attend a church or other religious service**			**Go out with someone you don’t know well**		
**< 3 months**	369	27.43	**< 3 months**	94	6.99
**3 to 6 months**	160	11.90	**3 to 6 months**	86	6.39
**6 to 12 months**	187	13.90	**6 to 12 months**	172	12.79
**> 1 year**	308	22.90	**> 1 year**	450	33.46
**Never again**	155	11.52	**Never again**	385	28.62
**Does not apply**	166	12.34	**Does not apply**	158	11.75
**Send kids to school. Camp or daycare**			**Stop routinely wearing a face covering**		
**< 3 months**	308	22.90	**< 3 months**	42	3.12
**3 to 6 months**	95	7.06	**3 to 6 months**	25	1.86
**6 to 12 months**	118	8.77	**6 to 12 months**	146	10.86
**> 1 year**	174	12.94	**> 1 year**	743	55.24
**Never again**	63	4.68	**Never again**	355	26.39
**Does not apply**	587	43.64	**Does not apply**	34	2.53
**Work in a shared office**			**Bring in the mail without precautions**		
**< 3 months**	459	34.13	**< 3 months**	252	18.74
**3 to 6 months**	115	8.55	**3 to 6 months**	104	7.73
**6 to 12 months**	131	9.74	**6 to 12 months**	160	11.90
**> 1 year**	185	13.75	**> 1 year**	442	32.86
**Never again**	116	8.62	**Never again**	339	25.20
**Does not apply**	339	25.20	**Does not apply**	48	3.57

## Discussion

Healthcare professionals and front-line workers are especially vulnerable in times of public health crisis and may be at risk for developing adverse psychological outcomes, particularly in low/middle-income Latin American countries [22]. As previously mentioned, a study conducted in Colombian otolaryngologists and allergists during the COVID-19 pandemic reported high rates of psychological outcomes [11, 23]. Our study describes the prevalence of mental health outcomes and associated factors of anxiety, depression, and stress in the general Colombian health care population during the COVID-19 pandemic. A total of 1345 health care professionals participated in this study including nurses, physicians, therapists, and dentists, among others, although nearly half of them were specialist doctors. Adverse psychological outcomes such as anxiety, depression, and stress were reported in 75,61%, 59,18%, and 53,09% of the study population, respectively, echoing the findings of previous studies conducted during the pandemic [23, 24]; as mentioned previously.

A systematic review published in 2020 by Vizheh et al., reported a prevalence of anxiety, depression, and stress among health care professionals of 67.55, 55.89, and 62.99%, respectively [5]. Similarly, a prior study in a Spanish healthcare population that reported a prevalence of psychological distress of up to 80.6% [25], which to date is the highest rate of this mental outcome in healthcare workers. However, we stand out that our results remain among the highest rates of these psychological outcomes compared to prior reports worldwide, which underscores the importance of urgent mental health strategies for healthcare workers. Up to 43.64% of our study population presented these 3 psychological disorders simultaneously, highlighting the importance of prevention and early interventions. However, anxiety, depression, and stress symptoms require psychiatric evaluation and confirmation since the questionnaires applied to the population cannot rule out that these symptoms could be related to adjustment disorders. Our data was collected 1 year after the pandemic started and despite there is a significant amount of scientific information about the mental health in healthcare workers in the beginning of the pandemic, prior authors state that depending on the trajectory of the pandemic the mental health symptoms on healthcare workers could intensify or reduce over time [26].

About the factors associated with the severity of anxiety, depression, and stress; a higher severity of these outcomes was found in women. Similarly, Gómez-Salgado et al reported that among 3801 adults living in Spain during COVID-19 confinement, women had higher levels of psychological discomfort [27]. Previous studies have reported that women can be twice as more prone to depression than men probably due to individual factors such as genetic, environmental, and cultural influences [28, 29]. Furthermore, prior authors describe that woman in healthcare are often victims of additional external factors for mental health outcomes such as bias and discrimination, disparaging or disrespectful comments, lack of career promotion, disparities in resources (including financial and administrative support), rewards, and reimbursement [30, 31]. Additionally, due to school closures during the pandemic, family caregivers may have had a greater burden of responsibilities. This scenario could explain the interaction found between being women and being single/widowed/divorced, since this population was less prone to depression. However, we also stand out that the variable “single/widowed/divorced” was associated with the presence of depression. Prior authors have described this relationship between marital status and depression: a higher prevalence of depression in separated or divorced individuals may be due to both an increased risk of marital disruption, and to the higher risk of this disorder in those with divorced or separated marital status [32]. Organizational support strategies are needed in these populations to increase wellbeing, improve resilience, provide protected time to participate in self-care activities, and convenient access to physical and mental health services should be prioritized.

Age was also negatively associated with psychological outcomes; older age could lead to a lower probability of severe forms of these conditions. Similarly, a prior study in healthcare workers in Iran during the pandemic reported a higher frequency of depression and anxiety in younger participants aged 30–39 years old compared to those aged 40 or older [33]. However, the presentation of depression in older adults may be significantly different to that in younger adults, since it can be present with the absence of an affective component [34, 35]. Therefore, despite depression is the most common mental disorder in older adults, it can be often under-diagnosed probably due to age-related biological and psychological factors, and comorbidities [34, 36]. Moreover, a higher incidence of chronic disease and using regular medication has been associated with higher levels of anxiety and an increased risk of negative mental outcomes [37]. Even though the mental health symptoms may show variations among older populations, higher rates of morbidity and mortality have been described, as well as increased healthcare utilization and economic costs. Further studies assessing these findings should be performed in older populations.

Furthermore, higher levels of anxiety were found in participants that expressed fear of a negative outcome such as death or negative sequelae which is similar to a prior study that included Otolaryngologists and allergists in Colombia [11]. Almost 88% reported fear of contagion, and over 92% were afraid of the possibility of infecting their family and/or friends, highlighting the sense of fear as key in developing adverse emotional symptoms. Although prior authors state that the increase in workload may generally be associated with increased stress [38], in this study the increase in workload may be also associated with anxiety and depression which has been previously described as global public health priority [39]. Moreover, in this study the decrease in consultations was associated with anxiety. This scenario may be related to a decrease in monthly income since an association was also found between a reduction in income during the pandemic and the presence of depression and stress. Prior studies have shown that a significant decrease in household income is associated with an increased risk of incident mood, or anxiety disorders [23, 40]. This highlights the importance of providing not only emotional support but preventive financial measures to healthcare workers who may experience monetary drawbacks due to lockdown policies. On a more personal level, endeavors such as engaging with family and friends or taking part in outdoor activities have been compromised by the sanitary situation, consequently shaping behavioral standards, and altering traditional support systems, and emotional outlets.

More than 28% of healthcare professionals surveyed, expressed that they would never go out again with someone they do not know well. More than 20% expressed that they will never exercise in a gym again, and most participants reported delaying non-urgent medical appointments to up to a year, highlighting the risks in terms of physical as well as emotional health. These changes in work patterns and daily life were also described in a prior study by Colombian otolaryngologists and allergists [9]. Radical changes in the daily life of healthcare workers may predispose them to the accelerated development of mental disorders. Prior authors have described the role of negative future-oriented cognitions in depression prone individuals [41]. However, this scenario could also be related to the timepoint when the questionnaires were applied, since this survey was performed during the highest rates of infection of the pandemic in Colombia. During this period the vaccination programs were still on the first phase and preliminary studies reported the challenges and limited access to vaccination in low- to middle-income countries [42]. Nevertheless, the long-term impact is still to be defined and may change as studies are conducted at different time points of the COVID-19 pandemic.

Among the strengths of this study, we stand out that the study sample included health workers from different professions, since epidemiologic research highlights the critical constructs of “representativeness” and its relationship with the “generalizability” of study results [43]. The current study may encounter limitations regarding the cross-sectional design of the study can display associations between the variables, but no causal relationships. The survey was completed only by 1360 out of 3512 people who registered to the questionnaire (38.72%), which could lead to a selection bias. We highlight that this study was performed prior to the vaccination campaigns in Colombia, therefore these symptoms could have significant differences compared to the results after vaccination.

Shining light upon the importance of mental health in healthcare workers is essential considering the high prevalence of adverse psychological outcomes raised by the COVID-19 pandemic. Preventive strategies include adequate occupational environments, financial support, and incentives to mitigate stresses from financial uncertainty for frontline and independent healthcare workers. Previous studies state that key strategies to reduce anxiety among healthcare workers include limitation of shift hours, clear communication, ensuring adequate rest areas, providing timely and appropriately tailored mental health support through hotline teams, media or multidisciplinary teams, and involving mental health professionals in follow-up, diagnose and provide early therapeutic interventions [44, 45]. A current systematic review stated that clear communication and support from the healthcare institutions, social support, and personal sense of control are protective factors for mental disorders on healthcare workers during pandemic outbreaks [46]. Further studies are essential to support preventive and therapeutic public health strategies to achieve early mental health prevention approaches, as well as therapeutic interventions in this population.

## Conclusions

During this COVID-19 pandemic, the frequency of anxiety, depression, and stress is high among this healthcare population compared to prior reports worldwide and the Colombian healthcare population. Anxiety, depression, and stress were more severe in women. Higher levels of anxiety, depression, and stress were found in participants that expressed fear of the possibility of a negative outcome (death, negative sequelae) due to COVID-19. The reduction in the number of consultations and surgery was also associated positively with anxiety severity, while the reduction in income during the pandemic was positively associated with higher levels of stress. Research on these psychological outcomes is needed in Latin America to achieve early mental health prevention approaches, as well as therapeutic interventions in this population. Psychological and/or psychiatric support without occupational stigmatization should be granted by the institutions.

## Data Availability

All data generated or analysed during this study are included in this published article [and its supplementary information files].

## References

[1] O’Leary VB, Ovsepian SV (2020). Severe Acute Respiratory Syndrome Coronavirus 2 (SARS-CoV-2). Trends Genet.

[2] Darmon M, Dumas G (2021). Anticipating outcomes for patients with COVID-19 and identifying prognosis patterns. Lancet Infect Dis.

[3] Danet A (2021). Psychological impact of COVID-19 pandemic in Western frontline healthcare professionals. A systematic review. Med Clin.

[4] Maunder RG, Lancee WJ, Balderson KE, Bennett JP, Borgundvaag B, Evans S (2006). Long-term psychological and occupational effects of providing hospital healthcare during SARS outbreak. Emerg Infect Dis.

[5] Vizheh M, Qorbani M, Arzaghi SM, Muhidin S, Javanmard Z, Esmaeili M. The mental health of healthcare workers in the COVID-19 pandemic: A systematic review. J Diabetes Metab Disord. 2020;19(2):1967–78. 10.1007/s40200-020-00643-9 PMC7586202 33134211

[6] Spoorthy MS, Pratapa SK, Supriya M (2020). Mental health problems faced by healthcare workers due to the COVID-19 pandemic–A review. Asian J Psychiatric.

[7] Rana W, Mukhtar S, Mukhtar S (2020). Mental health of medical workers in Pakistan during the pandemic COVID-19 outbreak. Asian J Psychiatr.

[8] Mental Health America (2021). The Mental Health of Healthcare Workers in COVID-19 | Mental Health America.

[9] Kock D (2009). A rapid review of the impact of COVID-19 on the mental health of healthcare workers: implications for supporting psychological well-being.

[10] Saragih ID, Tonapa SI, Saragih IS, Advani S, Batubara SO, Suarilah I (2021). Global prevalence of mental health problems among healthcare workers during the Covid-19 pandemic: A systematic review and meta-analysis. Int J Nurs Stud.

[11] Pérez-Herrera LC, Moreno-López S, Peñaranda D, Pérez-García IC, García E, Corredor-Rojas G (2021). Effect of the COVID-19 pandemic on the mental health, daily and occupational activities of otolaryngologists and allergists in Colombia: a national study. Int Forum Allergy Rhinol.

[12] Miranda MAM, Visbal JHW (2021). Mental health of colombian general practitioners during the pandemic: Reflections from a brief survey. Vol. 26, Hacia la Promocion de la Salud.

[13] Monterrosa-Castro A, Dávila-Ruiz R, Mejía-Mantilla A, Contreras-Saldarriaga J, Mercado-Lara M, Florez-Monterrosa C (2020). Occupational Stress, Anxiety and Fear of COVID-19 in Colombian Physicians. MedUNAB..

[14] Smallwood N, Karimi L, Bismark M, Putland M, Johnson D, Dharmage SC (2021). High levels of psychosocial distress among Australian frontline healthcare workers during the COVID-19 pandemic: a cross-sectional survey. General Psychiatry.

[15] Pappa S, Ntella V, Giannakas T, Giannakoulis VG, Papoutsi E, Katsaounou P (2020). Prevalence of depression, anxiety, and insomnia among healthcare workers during the COVID-19 pandemic: A systematic review and meta-analysis. Brain Behav Immun.

[16] Machin D, Campbell MJ, Tan SB, Tan SH (2011). Sample Size Tables for Clinical Studies (Google eBook).

[17] García-Campayo J, Zamorano E, Ruiz MA, Pardo A, Pérez-Páramo M, López-Gómez V (2010). Cultural adaptation into Spanish of the generalized anxiety disorder-7 (GAD-7) scale as a screening tool. Health Qual Life Outcomes.

[18] Saldivia S, Aslan J, Inostroza C (2019). Propiedades psicométricas del PHQ-9. Rev Med Chil.

[19] Campo-Arias A, Oviedo HC, Herazo E (2015). Escala de Estrés Percibido-10: Desempeño psicométrico en estudiantes de medicina de Bucaramanga, Colombia. Revista de la Facultad de Medicina.

[20] Escala LA, Percibido DEE, Trujillo HM (2007). Propiedades psicométricas de la versión española de la Escala de Estrés Percibido ( EEP ).

[21] Sanger-Katz M, Miller CC, Bui Q. When 511 Epidemiologists Expect to Fly, Hug and Do 18 Other Everyday Activities Again. https://www.nytimes.com/interactive/2020/06/08/upshot/when-epidemiologists-will-do-everyday-things-coronavirus.html. Accessed 30 Oct 2018.

[22] Peñaranda D, Perez-Herrera LC, Moreno-Lopez S, Peñaranda A (2021). COVID-19 Pandemic on Mental Health in Pediatric Otolaryngologists in Latin America. Otolaryngol Head Neck Surg.

[23] Padilla S, Calero-sierra I, Almazara C (2020). Mental health impact of COVID-19 pandemic on Spanish healthcare workers.

[24] Di Tella M, Romeo A, Benfante A, Castelli L (2020). Mental health of healthcare workers during the COVID-19 pandemic in Italy. J Eval Clin Pract.

[25] Gómez-Salgado J, Domínguez-Salas S, Romero-Martín M, Romero A, Coronado-Vázquez V, Ruiz-Frutos C (2021). Work engagement and psychological distress of health professionals during the COVID-19 pandemic. J Nurs Manag.

[26] Civantos AM, Byrnes Y, Chang C, Prasad A, Chorath K, Poonia SK (2020). Mental health among otolaryngology resident and attending physicians during the COVID-19 pandemic: National study. Head Neck.

[27] Gómez-Salgado J, Domínguez-Salas S, Rodríguez-Domínguez C, Allande-Cussó R, Romero-Martín M, Ruiz-Frutos C (2021). Gender perspective of psychological discomfort during COVID-19 confinement among Spanish adult population: a cross-sectional study. BMJ Open.

[28] Kuehner C (2003). Gender differences in unipolar depression: an update of epidemiological findings and possible explanations. Acta Psychiatr Scand.

[29] Kuehner C (2017). Why is depression more common among women than among men?. Lancet Psychiatry.

[30] Templeton K, Medical K, Bernstein CA, Einstein A, Sukhera J, Nora LM (2019). Gender-Based Di ff erences in Burnout : Issues Faced by Women Physicians. NAM Perspectives.

[31] Bacigalupe A, Martín U (2020). Gender inequalities in depression/anxiety and the consumption of psychotropic drugs: Are we medicalising women’s mental health?. Scand J Public Health.

[32] Bulloch AG, Williams JV, Lavorato DH, Patten SB (2009). The relationship between major depression and marital disruption is bidirectional. Depression Anxiety.

[33] Hassannia L, Taghizadeh F, Moosazadeh M, Zarghami M, Taghizadeh H, Dooki AF, et al. Anxiety and Depression in Health Workers and General Population During COVID-19 in IRAN: A Cross-Sectional Study. Neuropsychopharmacol Rep. 2021;41(1):40–9. Available from: 10.1002/npr2.12153. 10.1002/npr2.12153 PMC8182959 33369264

[34] Alexopoulos GS (2005). Depression in the elderly. Lancet.

[35] Fiske A, Wetherell JL, Gatz M (2009). Depression in Older Adults. Ann Rev Clin Psychol.

[36] Pocklington C, Gilbody S, Manea L, McMillan D (2016). The diagnostic accuracy of brief versions of the Geriatric Depression Scale: a systematic review and meta-analysis. Int J Geriatr Psychiatry.

[37] Mete B, Fırıncı B, Doğan E, Pehlivan E (2018). Depression and affecting factors in patients over 50 years of age: A cross-sectional study. J Surg Med.

[38] Portoghese I, Galletta M, Coppola RC, Finco G, Campagna M (2014). Burnout and workload among health care workers: the moderating role of job control. Saf Health Work.

[39] Søvold LE, Naslund JA, Kousoulis AA, Saxena S, Qoronfleh MW, Grobler C, et al. Prioritizing the Mental Health and Well-Being of Healthcare Workers: An Urgent Global Public Health Priority. Front Public Health. 2021;9 Available from: https://www.frontiersin.org/articles/10.3389/fpubh.2021.679397/full. 10.3389/fpubh.2021.679397 PMC8137852 34026720

[40] Sareen J, Afifi TO, KA MM, Asmundson GJ (2016). Relationship between household income and mental disorders: findings from a population-based longitudinal study. Arch Gen Psychiatry.

[41] Thimm J, Holte A, Brennen T, Wang CE. Hope and expectancies for future events in depression. Front Psychol. 2013:4, 470 Available from: https://www.frontiersin.org/article/10.3389/fpsyg.2013.00470. 10.3389/fpsyg.2013.00470 PMC3721024 23898314

[42] Tagoe ET, Sheikh N, Morton A, Nonvignon J, Sarker AR, Williams L, et al. COVID-19 Vaccination in Lower-Middle Income Countries: National Stakeholder Views on Challenges, Barriers, and Potential Solutions. Front Public Health. 2021:9 Available from: https://www.frontiersin.org/article/10.3389/fpubh.2021.709127. 10.3389/fpubh.2021.709127 PMC8377669 34422750

[43] Kukull WA, Ganguli M (2012). Generalizability: the trees, the forest, and the low-hanging fruit. Neurol Int.

[44] Ramanathan K, Antognini D, Combes A, Paden M, Zakhary B, Ogino M, et al. Mental health care for medical staff in China during the COVID-19 outbreak. Lancet Psychiatry. 2020;7(4):e15–e16. 10.1016/S2215-0366(20)30078-X PMC7129426 32085839

[45] Blake H, Bermingham F, Johnson G, Tabner A (2020). Mitigating the psychological impact of covid-19 on healthcare workers: A digital learning package. International Journal of Environmental Research and Public Health, núm 17. Recuperado el 16 de Agosto del 2020. Int J Environ Res Public Health.

[46] De Brier N, Stroobants S, Vandekerckhove P, De Buck E (2020). Factors affecting mental health of health care workers during coronavirus disease outbreaks (SARS, MERS & COVID-19): A rapid systematic review. PLoS ONE.

